# Identification and functional characterisation of *N*-linked glycosylation of the orphan G protein-coupled receptor Gpr176

**DOI:** 10.1038/s41598-020-61370-y

**Published:** 2020-03-10

**Authors:** Tianyu Wang, Shumpei Nakagawa, Takahito Miyake, Genzui Setsu, Sumihiro Kunisue, Kaoru Goto, Akira Hirasawa, Hitoshi Okamura, Yoshiaki Yamaguchi, Masao Doi

**Affiliations:** 1 Department of Systems Biology, Graduate School of Pharmaceutical Sciences, Kyoto University, Sakyō-ku Kyoto, 606-8501 Japan; 2 Department of Genomic Drug Discovery Science, Graduate School of Pharmaceutical Sciences, Kyoto University, Sakyō-ku Kyoto, 606-8501 Japan; 3 Present Address: Laboratory of Molecular Brain Science, Graduate School of Pharmaceutical Sciences, Kyoto University, Sakyō-ku Kyoto, 606-8501 Japan

**Keywords:** Circadian rhythms and sleep, Post-translational modifications, Hormone receptors, Glycosylation, Mutation

## Abstract

G-protein-coupled receptors (GPCRs) are important drug targets with diverse therapeutic applications. However, there are still more than a hundred orphan GPCRs, whose protein functions and biochemical features remain unidentified. *Gpr176* encodes a class-A orphan GPCR that has a role in circadian clock regulation in mouse hypothalamus and is also implicated in human breast cancer transcriptional response. Here we show that Gpr176 is *N*-glycosylated. Peptide-*N*-glycosidase treatment of mouse hypothalamus extracts revealed that endogenous Gpr176 undergoes *N*-glycosylation. Using a heterologous expression system, we show that *N*-glycosylation occurs at four conserved asparagine residues in the N-terminal region of Gpr176. Deficient *N*-glycosylation due to mutation of these residues reduced the protein expression of Gpr176. At the molecular function level, Gpr176 has constitutive, agonist-independent activity that leads to reduced cAMP synthesis. Although deficient *N*-glycosylation did not compromise this intrinsic activity, the resultant reduction in protein expression was accompanied by attenuation of cAMP-repressive activity in the cells. We also demonstrate that human GPR176 is *N*-glycosylated. Importantly, missense variations in the conserved *N*-glycosylation sites of human GPR176 (rs1473415441; rs761894953) affected *N*-glycosylation and thereby attenuated protein expression and cAMP-repressive activity in the cells. We show that *N*-glycosylation is a prerequisite for the efficient protein expression of functional Gpr176/GPR176.

## Introduction

G-protein-coupled receptors (GPCRs) are the largest family of cell-surface receptors and are the therapeutic targets of nearly a third of clinically marketed drugs^1,2^. Despite their importance, more than one hundred human GPCRs remain poorly characterised due to the lack of useful information on their ligands^3^. Included among these so-called orphan GPCRs is GPR176, which is predicted to be a 56-kDa seven-transmembrane protein of class A GPCR with potential sites for *N*-glycosylation.

*GPR176* (also known as *HB-954*) was initially cloned by Hata *et al*. from a human brain cDNA library^4^. In the mouse brain, *Gpr176* mRNA levels are predominantly high in the suprachiasmatic nucleus of the hypothalamus (SCN)^5^, the principal circadian pacemaker in mammals, and knockout studies have shown that *Gpr176* is required to set the pace of circadian rhythm in behaviour^5^. This gene is also expressed in other tissues than the brain^4^ and was reported to be involved in the anacardic acid-induced transcriptional response of human breast cancer cells^6^. Gpr176 couples to Gz, a subtype of Gi/o, and even in the absence of a known ligand, Gpr176 possesses an agonist-independent constitutive activity that leads to reduced cAMP synthesis^5,7^. At the amino acid sequence level, Gpr176 contains five extracellular potential sites for *N*-glycosylation (Asn-X-Ser/Thr, where X is any amino acid except for Pro); one is located in the third extracellular loop (ECL3) and all other four are located in the N-terminal region. However, whether these sites receive *N*-glycosylation remains unknown.

Not surprisingly, knowledge regarding the presence and potential functional role(s) of the *N*-glycosylation of orphan GPCRs has been particularly sparse relative to that of receptors with known ligands. Interestingly, previous studies based on receptors with known ligands demonstrated that the functional role(s) of *N*-glycosylation varied depending on the type of GPCRs tested^8–26^. *N*-glycosylation of GPCRs may be important for their structural maturation, cell surface expression, ligand binding, and downstream signal transduction^8–27^. Because of the nonredundant functional nature of *N*-glycosylation, its role for the orphan Gpr176 must be determined empirically. It is also known that *N*-glycosylation occurs co-translationally—that is, during protein synthesis—in the lumen of the endoplasmic reticulum (ER)^28–32^.

Here, we describe the identification and characterisation of *N*-linked glycosylation of the orphan receptor Gpr176. We show that Gpr176 is *N*-glycosylated *in vivo* in the mouse SCN. Using a heterologous expression system, we show that *N*-glycosylation occurs at four conserved asparagine residues in the N-terminal region of mouse Gpr176 (N4, N11, N17, N26). Prevention of *N*-glycosylation by the mutation of these sites led to a drastic reduction in Gpr176 protein expression. Non-glycosylated mutant proteins were mostly retained in the ER, suggesting a problem during protein biosynthesis. At the molecular level, deficient *N*-glycosylation did not impair the constitutive activity of Gpr176. Nevertheless, the reduction in Gpr176 protein expression caused by lack of *N*-glycosylation led to reduced total cAMP-repressive activity in the cells. Finally, in an attempt to extend our findings to humans, we analysed polymorphic variations of human GPR176. In this study, five reported rare nonsynonymous SNPs (single nucleotide polymorphisms; rs1473415441, rs1478819979, rs761894953, rs1293261954, and rs556899221) that are located in the evolutionarily conserved *N*-linked glycosylation sites were analysed. We show that *N*-glycosylation is a prerequisite for the maximal protein expression of functional GPR176 (Gpr176).

## Results

### Gpr176 undergoes ***N***-glycosylation *in vivo*

**Figure 1:**
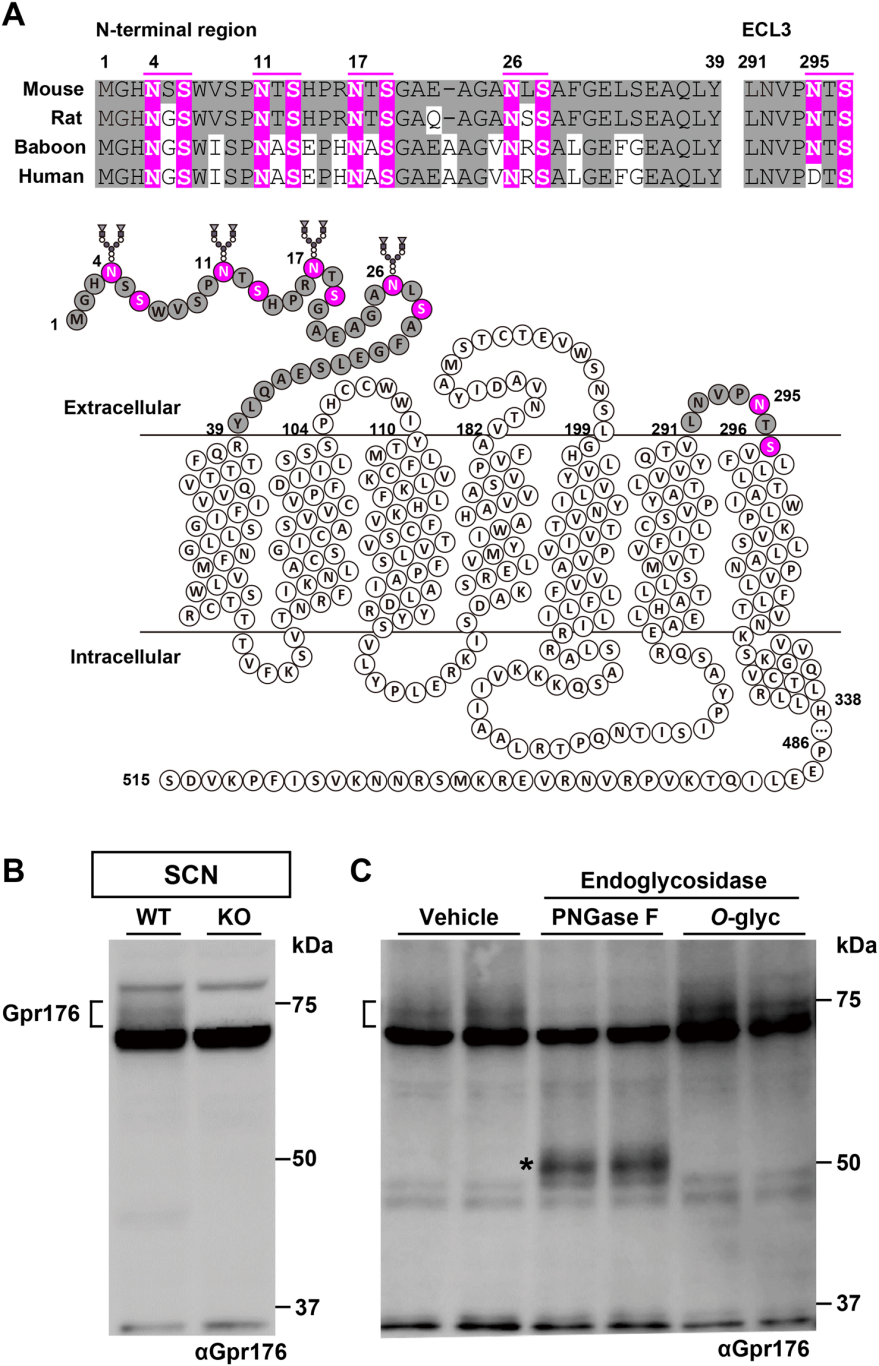
Gpr176 is an *N*-glycosylated GPCR. (**A**) Amino acid sequence conservation of the potential *N*-glycosylation sites in the N-terminal and extracellular loop 3 (ECL3) region of mouse, rat, baboon, and human Gpr176. Sequences that match the consensus *N*-linked glycosylation motif (N-X-S/T, where X is not P) are highlighted in magenta in the alignments as well as in the snake-plot representation of mouse Gpr176. (**B,C**) Immunoblot of mouse hypothalamus SCN cell extracts from WT and *Gpr176*^*−/−*^ mice for Gpr176 (**B**). The WT extracts were treated with vehicle or either PNGase F or *O*-glycosidase (*O*-glyc) and immunoblotted for Gpr176 (**C**). Two independent biological replicates per condition were loaded in (**C**). Note that a broad, heterogeneous band with an apparent molecular mass of 75 kDa was detectable in only the WT extracts. The asterisk indicates the position of deglycosylated Gpr176.

Amino acid sequences that match the consensus *N*-glycosylation motif (Asn-X-Ser/Thr, where X is not Pro) in Gpr176 (Fig. 1A) prompted us to ask whether Gpr176 is glycosylated *in vivo*.

Western blotting of endogenous Gpr176 in the mouse hypothalamic SCN extract using an antibody against Gpr176 revealed a broad band migrating at around 75 kDa, which was down shifted toward ~50 kDa following Peptide-*N*-glycosidase F (PNGase F) treatment, consistent with its predicted molecular mass and *N*-linked glycosylation (Fig. 1B,C). *O*-glycosidase treatment, which digests *O*-linked sugars, had little or no apparent effect on the SDS-PAGE mobility of Gpr176. The absence of the proteins migrating at around 75 kDa in the SCN extract prepared from *Gpr176*^−/−^ mice (Fig. 1B) confirmed the specificity of the antibody used, which was raised against the C-terminal intracellular region of mouse Gpr176.

### *N*-glycosylation occurs at the N-terminal region of Gpr176

**Figure 2:**
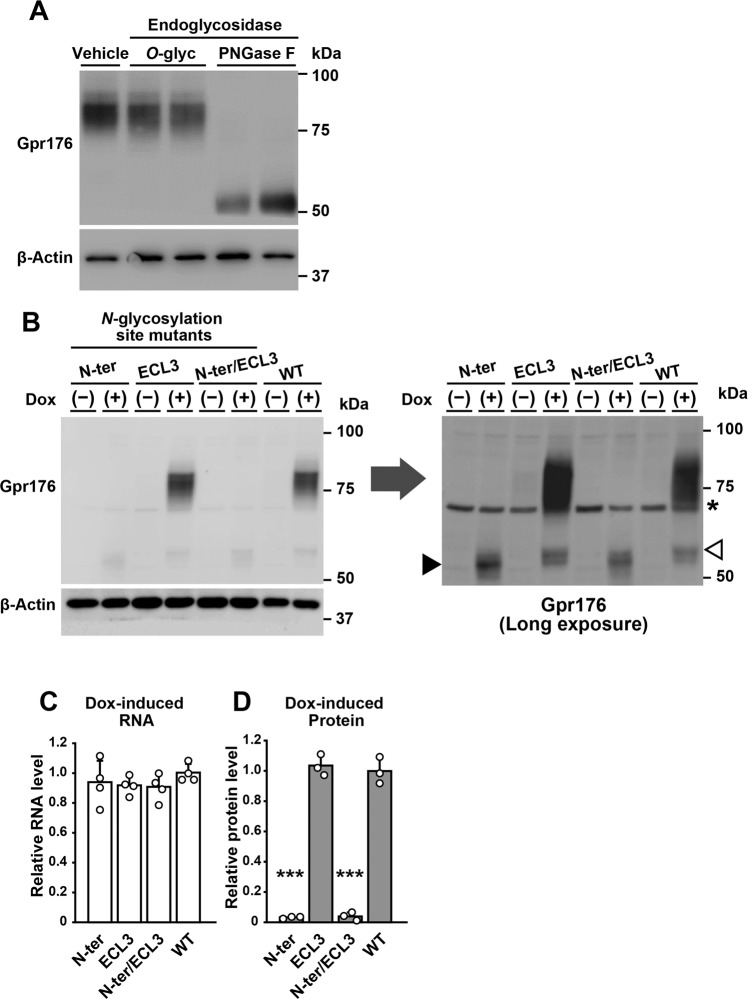
*N*-glycosylation at the N-terminal region is required for the optimal expression of Gpr176. (**A**) Gpr176 is *N*-glycosylated in Flp-In TREx293 cells. Dox-treated Flp-In TREx293-Gpr176 (tet-on) cell extracts were treated with PNGase F or *O*-glyc and immunoblotted for Gpr176 (upper) and β-Actin (lower). (**B**) Immunoblots of Dox-treated (+) and untreated (−) Flp-In TREx293 (tet-on) cells expressing WT Gpr176 and the respective mutants for potential *N*-linked glycosylation sites: N-ter (N4, 11, 17, 26Q), ECL3 (N295Q), and N-ter/ECL3 (N4, 11, 17, 26, 295Q). The same membrane exposed for a longer time is shown on the right. A closed arrowhead indicates the position of the proteins with an N-ter mutation. An open arrowhead points to a minor fraction of Dox-induced WT proteins. Asterisk, nonspecific bands. (**C,D**) Dox-induced mRNA (**C**) and protein (**D**) expression levels of WT Gpr176 and the N-ter, ECL3, and N-ter/ECL3 Gpr176 mutants. Relative *Gpr176* mRNA levels were determined by qRT-PCR and normalized to the expression levels of the gene encoding the ribosomal phosphoprotein P0. Values are the means ± s.d. (*n* = 4). For protein levels, relative band intensities in blots exposed for a longer time (**B**) were determined using densitometry. ***P < 0.0001 versus WT, one-way ANOVA with Bonferroni *post hoc* test. Values are the means ± s.d. (*n* = 3).

Similar to the endogenous Gpr176, recombinant mouse Gpr176 protein that was expressed in human embryonic kidney (HEK)293-derived Flp-In T-REx 293 cells displayed an apparent molecular mass of ~75 kDa and was shifted down to ~50 kDa after PNGase F treatment (Fig. 2A), indicating that exogenously expressed Gpr176 underwent *N*-glycosylation in a manner similar to that *in vivo*.

Site-directed single amino acid mutation of each potential *N*-glycosylation site implicated that all four consensus sites in the N-terminal region of Gpr176 (N4, N11, N17, and N26) are glycosylated (see Supplementary Fig. 1; while N11 seems less involved in contributing to the overall Gpr176 glycosylation, all tested mutants exhibit a small but noticeable shift in PAGE). Thus we generated mutant Gpr176 protein harbouring simultaneous Asn (N) to Gln (Q) substitutions of the N-terminal consensus *N*-glycosylation sites (N4Q;N11Q;N17Q;N26Q), designated hereafter as N-ter mut. N-ter mut and wild-type (WT) Gpr176 were expressed in Flp-In TREx293 cells in a doxycycline (Dox)-inducible manner. Immunoblots of Dox-treated (+) and -untreated (−) cells revealed that the apparent molecular mass of the mutant Gpr176 protein that appeared after Dox treatment (indicated by a solid arrowhead in Fig. 2B) was approximately 50 kDa, akin to the PNGase F-treated WT Gpr176.

In addition to this change in size, we noticed that the removal of the N-terminal glycosylation sites caused a drastic reduction in the total protein expression level of Gpr176. At the mRNA level, the expression of N-ter mut was normal (Fig. 2C), and β-actin displayed similar expression levels in all samples (Fig. 2B). However, the mean protein expression level of N-ter mut was reduced to less than 10% of that of WT (Fig. 2D), necessitating extended exposure for immunoblot detection (Fig. 2B). We found the apparent molecular mass of the other mutant that lacked the potential *N*-glycosylation site in the third extracellular loop (ECL3) to be ~75 kDa and its protein abundance almost indistinguishable from that of WT Gpr176 (Fig. 2B–D), while combined mutation of the site in ECL3 and the N-terminal sites reproduced the reduction in size and abundance of Gpr176 (Fig. 2B–D, N-ter/ECL3). These results indicate that N-terminal *N*-glycosylation is required for the proper protein expression of Gpr176.

Additionally, we noticed that in cells in which Gpr176 was overexpressed, a minor fraction of the WT proteins migrated at approximately 50 kDa (Fig. 2B; open arrowhead), which possibly reflects the presence of immature or less glycosylated proteins, as reported for other GPCRs and non-GPCR glycoproteins that have been overexpressed^33–36^.

### *N*-glycosylation is a prerequisite for the proper cell surface expression of Gpr176

**Figure 3:**
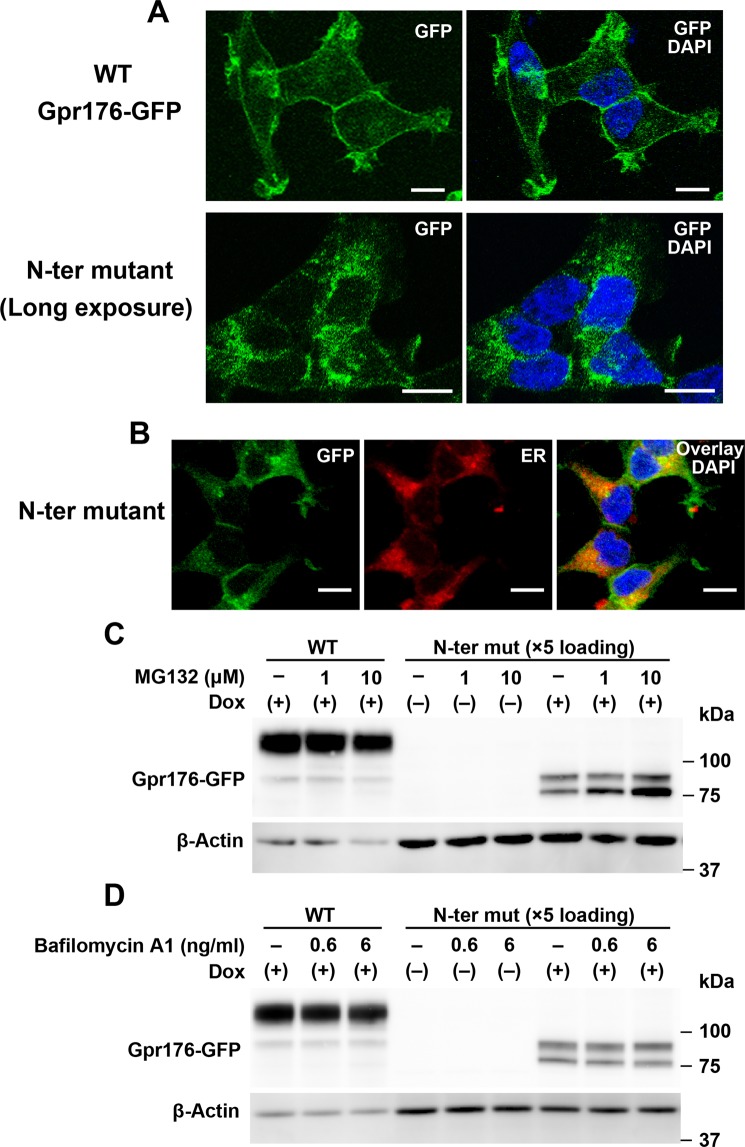
The prevention of *N*-glycosylation leads to reduced cell surface expression of Gpr176. (**A**) Representative confocal images of Flp-In TREx293 cells expressing WT (upper) or N-ter mut Gpr176-GFP (lower). Nuclei were stained with DAPI. The merged image is a combined image of GFP (green) and DAPI (blue). Gamma levels were adjusted over the whole image to optimize the appropriate GFP signals. The images are representative of three experiments. Scale bars, 10 μm. (**B**) Confocal images showing intracellular accumulation of N-ter mut Gpr176-GFP (green) in ER. Cells were stained with ER-Tracker (red). The merged image is a combined image with DAPI (blue). Cells are representative of a population with independent experiments repeated four times. Scale bars, 10 μm. (**C,D**) Immunoblots examining the effect of MG132 (**C**) and bafilomycin A1 (**D**) treatment on the expression of WT and N-ter mut Gpr176-GFP. Cells were treated with MG132 (**C**) or bafilomycin A1 (**D**) at the indicated concentrations for 6 h. The protein extracts of N-ter mutant cells were loaded at five-fold higher levels compared to those of WT cells to increase the sensitivity of protein detection. Note that mutant expression was partially restored by MG132 (**C**) but not by bafilomycin A1 (**D**), while under both conditions, WT expression was nearly unchanged.

To test the effect of deficient *N*-glycosylation on the subcellular localization of Gpr176, we examined a Flp-In TREx293 cell line expressing GFP-fused Gpr176 (Fig. 3). GFP was fused to the C-terminal end of Gpr176. Confocal microscopy revealed that the WT Gpr176-GFP fluorescence was distributed mostly in the plasma membrane (Fig. 3A), congruent with the reported cell-membrane immunolocalization of untagged Gpr176^5^. Attenuated N-ter mut protein expression was also observed for the mutant protein fused to GFP (Supplementary Fig. 2). The corresponding GFP signals were therefore intensified when imaging the subcellular localization of the mutant. We found that, under these conditions, the fluorescent signals of the N-ter mut were more intense in a perinuclear compartment corresponding to the ER than in the plasma membrane (see Fig. 3A,B and Supplementary Fig. 3), suggesting a problem during the process of protein synthesis and maturation in the ER. Total protein levels of mutant Gpr176 were partly restored by treatment of the cells with MG132 (a proteasome inhibitor) but not with bafilomycin A1 (a lysosomal inhibitor) (Fig. 3C,D). Homologous results were observed in experiments using untagged Gpr176 (Supplementary Fig. 4). Our results indicate that a majority of the non-glycosylatable Gpr176 proteins are retained in ER and degraded in part through a proteasomal pathway.

### Attenuated N-ter mut expression is accompanied by reduced total activity of Gpr176

**Figure 4:**
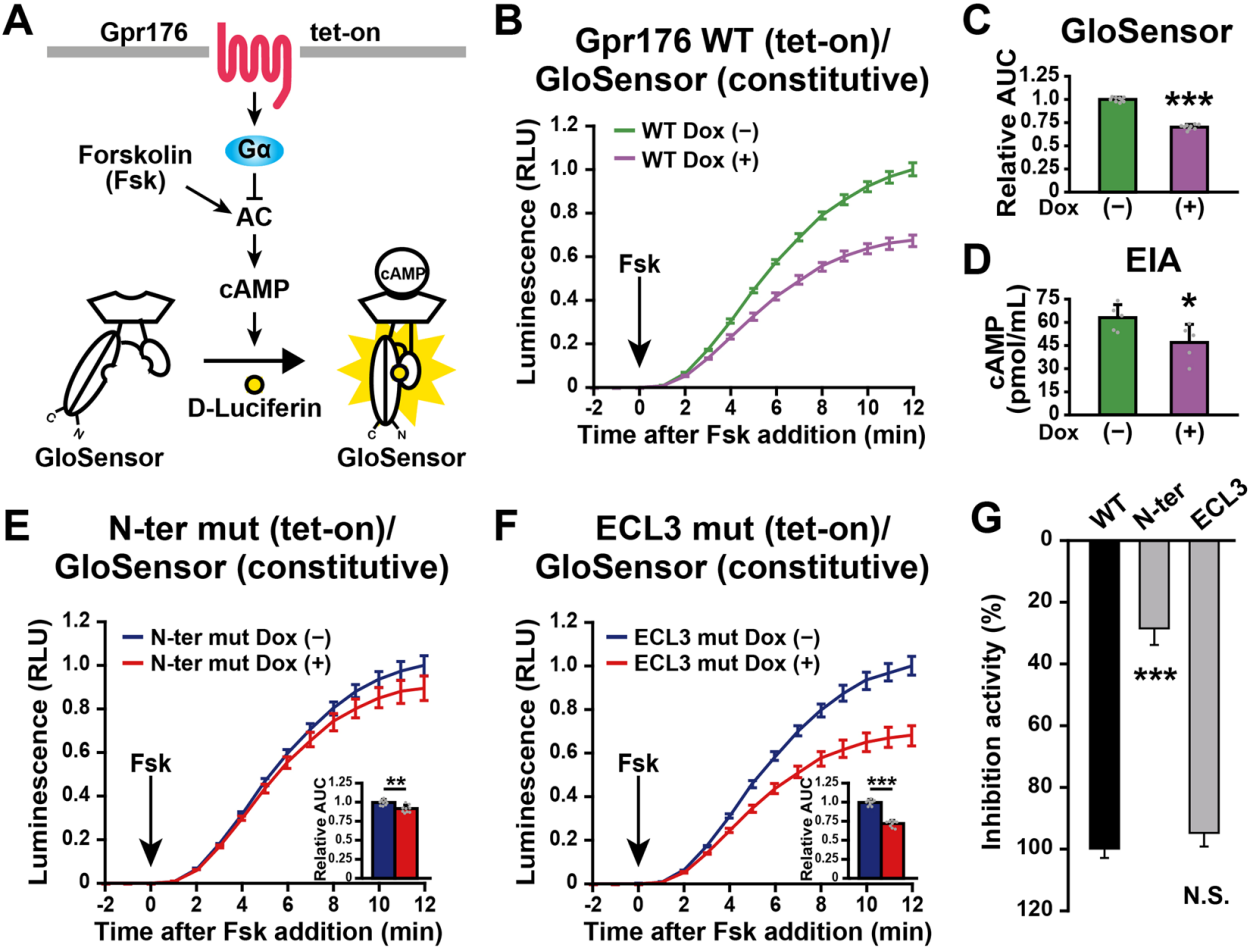
Attenuated expression of the glycosylation mutant results in reduced total activity of Gpr176. (**A**) Experimental design based on Flp-In TREx293-Gpr176 (tet-on)/GloSensor (constitutive) cells. (**B**) Forskolin (Fsk)-induced cAMP GloSensor luciferase activity traces in Dox-treated (+) and untreated (−) Flp-In TREx293-Gpr176 (tet-on)/GloSensor (constitutive) cells. The arrow indicates the start of Fsk treatment. RLU, relative light units. Values (means ± s.d., *n* = 9 for each data point) are plotted relative to the average peak value obtained in untreated cells. (**C**) Relative area under the curve (AUC) of luminescence values in (**B**). Light emissions were integrated and normalized with those of the untreated control. ***P < 0.0005, two-tailed unpaired *t*-test (*n* = 9 for each condition). Error bars indicate s.d. (**D**) Intracellular cAMP concentrations determined by cAMP enzyme immunoassay (EIA). *P < 0.05, two-tailed unpaired *t*-test (*n* = 5 for each conditio*n*). Error bars indicate s.d. (**E**) GloSensor activity traces in Dox-treated (+) and untreated (−) Flp-In TREx293 N-ter mut Gpr176 (tet-on)/GloSensor (constitutive) cells. Data are the means ± s.d. (*n* = 9). Insets indicate relative AUC values (means ± s.d., *n* = 9; **P < 0.001, two-tailed unpaired *t*-test). (**F**) GloSensor traces in Dox-treated (+) and untreated (−) Flp-In TREx293 ECL3 mut Gpr176 (tet-on)/GloSensor (constitutive) cells. Values are the means ± s.d. (*n* = 9). Insets indicate relative AUC values (means ± s.d., *n* = 9; ***P < 0.0005, two-tailed unpaired *t*-test). (**G**) Percent cAMP-inhibitory activities of N-ter and ECL3 mut Gpr176 relative to that of WT Gpr176. The AUC was used to evaluate the rate of cAMP inhibition. The values are plotted as a percent relative to WT Gpr176 (100%). Data are the means ± s.e.m. of nine replicates each. ***P < 0.0005 versus WT, one-way ANOVA with Bonferroni *post hoc* test. N.S., not significant.

In agreement with previous reports^5,7^, Dox-induced expression of WT Gpr176 led to the attenuation of forskolin (Fsk)-induced intracellular cAMP accumulation (Fig. 4), confirming that this orphan receptor has constitutive, agonist-independent activity. Changes in the intracellular cAMP level were assayed using either a luciferase-based cAMP biosensor (GloSensor 22 F) that was stably incorporated into the cells (Fig. 4A) or a cAMP enzyme-immunoassay (EIA). As observed in Fig. 4B–D, regardless of the type of assay method used, the amplitude of cAMP accumulation was significantly attenuated by ~30% in Dox-treated (+) cells versus untreated (−) cells (*P* < 0.001 for the GloSensor assay, *P* < 0.05 for EIA, two-tailed unpaired *t*-test), suggesting that the GloSensor assay and EIA were equivalent in their ability to detect Gpr176-derived cAMP-repressive activity in the cells (see also Supplementary Fig. 5).

Using the GloSensor assay, we then evaluated the functional consequence of attenuated protein expression of glycosylation-deficient N-ter mut Gpr176. We observed that cells expressing N-ter mut still exhibited measurable cAMP-repressive activity, albeit with a significantly diminished amplitude compared to that of cells expressing WT Gpr176 (see Fig. 4E,G, *P* < 0.0005, WT versus N-ter mut, one-way ANOVA, Bonferroni test). Although it may be argued that *N*-glycosylation has a direct effect on the molecular function of Gpr176, there is another possible interpretation of this result. Since the activity was measured in cells and its activity levels were correlated to Gpr176 protein expression levels in cells (see Supplementary Fig. 6), the observed decrease in cAMP-repressive activity might reflect the reduced protein expression of the mutant in cells. We also showed that, unlike cells expressing N-ter mut, cells expressing the ECL3 mutant had normal cAMP-repressive activity that was indistinguishable from that of cells expressing WT Gpr176 (Fig. 4F,G).

### *N*-glycosylation is not essential for the basal activity of Gpr176

**Figure 5:**
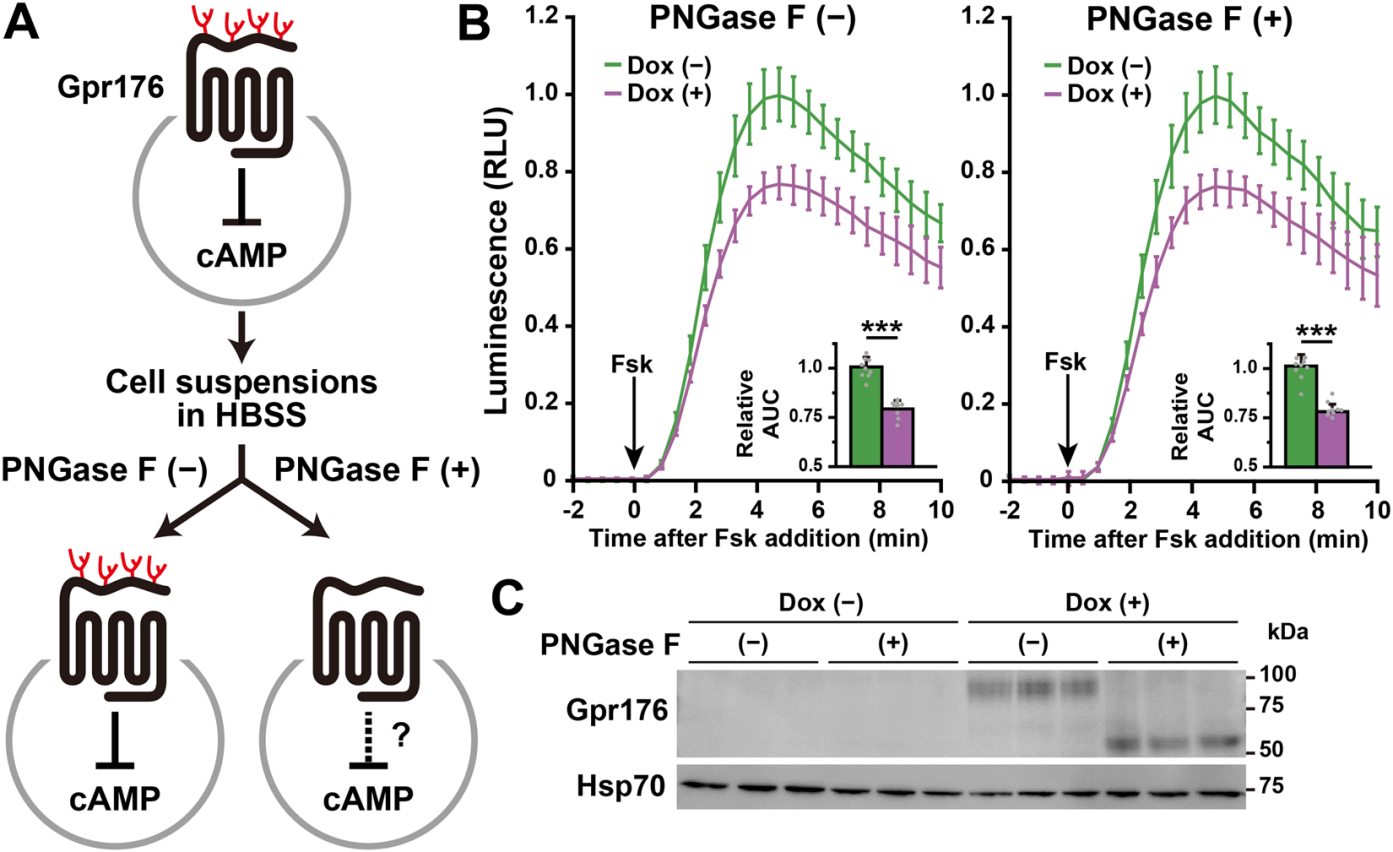
PNGase F-treated Gpr176 retains its cAMP-repressive activity. (**A**) Schematic experimental design for the glycosidase-treated cell suspension GloSensor assay. Dox-treated (+) and untreated (−) cells were resuspended in HBSS with or without PNGase F for 1 h before the GloSensor cAMP assay. (**B**) Fsk-induced GloSensor activity traces in PNGase F-treated and untreated Flp-In TREx293-Gpr176 (tet-on)/GloSensor (constitutive) cell suspensions. The arrow indicates the start of Fsk treatment. RLU, relative light units. Values (means ± s.d., *n* = 9 for each data point) are plotted relative to the average peak value obtained in Dox-untreated cells. Insets indicate relative AUC values (means ± s.d., *n* = 9; ***P < 0.0005, two-tailed unpaired *t*-test). (**C**) Immunoblots of representative PNGase F-treated and untreated Flp-In TREx293-Gpr176 (tet-on)/GloSensor (constitutive) cells. The cell suspension samples were immunoblotted for Gpr176 (upper) and Hsp70 (lower). Hsp70 served as a loading control.

Because prevention of the *N*-glycosylation of Gpr176 resulted in a drastic reduction in protein expression, site-directed mutagenesis may not be appropriate to address the role of *N*-glycosylation in the molecular function of Gpr176. To circumvent this inherent problem, we turned to a different approach. As shown in Fig. 5A, cells underwent deglycosylation treatment before Gpr176 activity was assayed. Freshly dissociated Dox-treated (+) and untreated (−) cell suspensions were treated with or without PNGase F for 1 h. Following enzymatic depletion of *N*-linked glycosylation, cells were immediately assayed for forskolin-induced changes in GloSensor activity (Fig. 5B). Western blotting (Fig. 5C) demonstrated that PNGase F treatment caused a down-shift of the Gpr176 band but did not alter the protein expression level of Gpr176. Under these conditions, we found that deglycosylated Gpr176 still had an essentially unimpaired ability to reduce cAMP accumulation (*P* < 0.0005, for both PNGase F (−) and (+) conditions, see Fig. 5B). These data suggest that *N*-glycosylation is not an absolute requirement for the agonist-independent constitutive function of Gpr176.

### *N*-glycosylation of human GPR176

**Figure 6:**
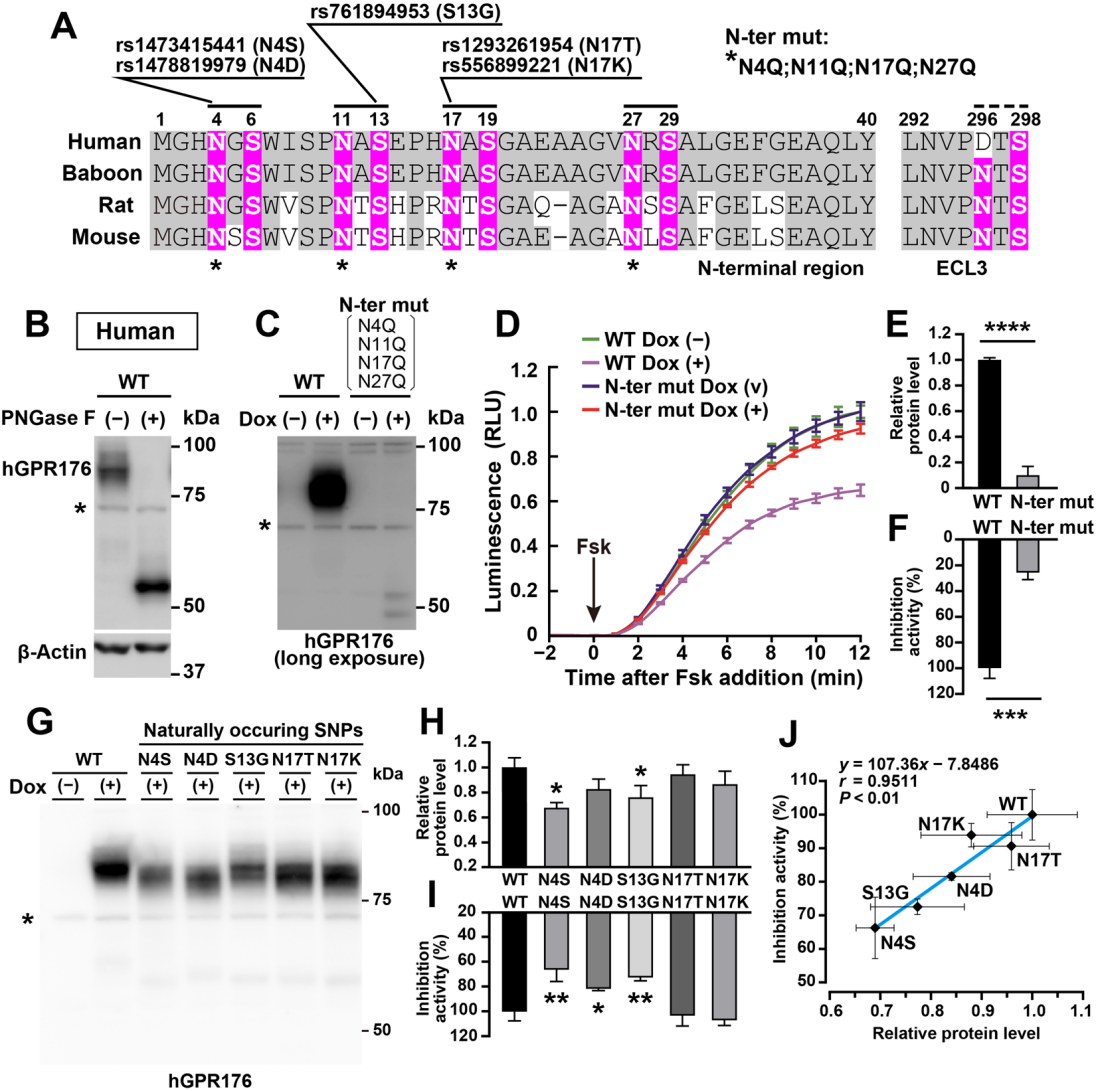
*N*-glycosylation of hGPR176 and its naturally occurring nonsynonymous variants. (**A**) Reported rare nonsynonymous SNPs of hGPR176. Note that the S13G variant disrupts the N-X-S triplet sequence required for glycosylation at N11. (**B**) Immunoblot of Dox-treated Flp-In TREx293-hGPR176 (tet-on) cells with or without PNGase F treatment. Asterisk, nonspecific bands. (**C**) Immunoblots of Dox-treated (+) and untreated (−) Flp-In TREx293 (tet-on) cells for WT and N-ter mut (N4Q;N11Q;N17Q;N27Q) hGPR176. (**D**) GloSensor activity traces in Dox-treated (+) and untreated (−) Flp-In TREx293 WT and N-ter mut hGPR176 (tet-on) cells. Values are the means ± s.d. (*n* = 3 for each data point). (**E**) Relative protein expression levels of Dox-induced WT and N-ter mut hGPR176. Values are the means ± s.d. (*n* = 3 for each) of the relative band intensities in (**C**). ****P < 0.0001, two-tailed unpaired *t*-test. (**F**) Percent cAMP-inhibitory activity of N-ter mut hGPR176 relative to that of WT hGPR176. The AUC in (**D**) was used to evaluate the rate of cAMP inhibition. Data are the means ± s.d. of three replicates each. ***P < 0.001, two-tailed unpaired *t*-test. (**G**) SDS-PAGE/immunoblot profiles of naturally occurring hGPR176 variants. We generated Flp-In TREx293 tet-on stable cell lines expressing each variant with the constitutive expression of GloSensor. (**H**) Relative protein expression levels of WT hGPR176 and respective variants of hGPR176. Values are the means ± s.d. (*n* = 3 for each). *P < 0.05, versus WT, one-way ANOVA with Bonferroni *post hoc* test. (**I**) Percent cAMP-inhibitory activities of respective hGPR176 variants relative to that of WT hGPR176. Data are the means ± s.d. of three replicates each. **P < 0.001, *P < 0.01, versus WT, one-way ANOVA with Bonferroni *post hoc* test. (**J**) Relationship between protein expression level (*x* axis) and inhibition activity (*y* axis) of WT and respective variants of hGPR176. Values are the means ± s.d. (*n* = 3 for each data point). Correlation coefficient (*r*) and *P* value were calculated by Pearson product moment correlation coefficient analysis (*r* = 0.9511, *P* < 0.01).

Finally, we analysed whether *N*-glycosylation of Gpr176 was also conserved in humans. Human GPR176 (hGPR176) has four conserved triplet sequences (N-X-S, X ≠ P) at its N-terminal extracellular region, but not in ECL3 (Fig. 1A), and rare missense variants have been reported for 3 of the 4 N-terminal sequons (Fig. 6A; dbSNP, https://www.ncbi.nlm.nih.gov/snp/). These mutations have the potential to influence *N*-glycosylation at positions 4, 11, and 17; however, there is no SNP reported for N27.

As observed for mouse Gpr176, recombinant hGPR176 protein expressed in Flp-In T-REx 293 cells displayed an apparent molecular mass of ~75 kDa and was downshifted to ~50 kDa after PNGase F treatment (Fig. 6B). Consistent with results from mouse Gpr176, simultaneous mutation of the four conserved asparagine residues (N4Q;N11Q;N17Q;N27Q), i.e., the human version of N-ter mut, led to an attenuation of protein expression (Fig. 6C,E) as well as reduced total cAMP-repressive activity in the cells (Fig. 6D,F) (*P* < 0.0001 for protein expression; *P* < 0.001 for activity, two-tailed unpaired *t*-test). In hGPR176, there are 5 rare nonsynonymous SNPs (minor allele frequency < 0.01%) that induce the amino acid replacements of N4D, N4S, S13G, N17T, and N17K. To test the effect of these naturally occurring SNPs on hGPR176 expression and activity, the respective variants were introduced into Flp-In TREx293 cells (Fig. 6G). In contrast to the SDS-PAGE mobility of N-ter mut, each SNP had only a mild effect on the SDS-PAGE mobility of hGPR176 (Fig. 6G), consistent with the idea that these naturally occurring SNPs only affect one possible *N*-glycosylation site in hGPR176. Likewise, overall, these SNPs did not induce a profound reduction in protein expression compared to N-ter mut. Nevertheless, to a lesser extent, the two distinct SNPs that encode N4S (rs1473415441) and S13G (rs761894953) were found to induce a small but statistically significant reduction in both protein expression and cAMP-repressive activity in the cells (*P* < 0.05 for protein expression, *P* < 0.001 for activity, one-way ANOVA, Bonferroni test, see Fig. 6G–I and Supplementary Fig. 7). Similarly, the variant encoding N4D caused a significant attenuation in activity (*P* < 0.01; see Fig. 6I and Supplementary Fig. 7); however, while there was a trend toward decreased protein expression, this decrease was not significant (Fig. 6H). On the other hand, neither the protein expression nor the activity of N17T and N17K showed a significant change (Fig. 6H,I), suggesting that the deficits in protein levels are correlated to signalling activity deficits of the mutants (Pearson correlation coefficient, *r* = 0.9511, *P* < 0.01, Fig. 6J). These phenotypic differences between SNPs provide a basis for understanding the *N*-glycosylation-related polymorphisms of hGPR176.

## Discussion

*N*-glycosylation is one of the most common posttranslational modifications of membrane protein GPCRs, yet literature regarding the presence and potential functional roles of *N*-glycosylation of orphan GPCRs remains particularly sparse. In the present study, we identified and characterised *N*-glycosylation of the orphan GPCR Gpr176. Importantly, the functional significance of *N*-glycosylation differs between GPCRs (see ref. 8–27). In the case of Gpr176, *N*-glycosylation is required for its efficient protein expression. However, this does not necessarily hold true for other GPCRs. The orphan GPCR Gpr61 was reported to be *N*-glycosylated^27^, but *N*-glycosylation is not vital for its expression^27^. Similarly, it was shown that the expression levels of the α_1_-adrenergic receptor^8^, M_2_ muscarinic receptor^9^, histamine H_2_ receptor^10^, vasopressin V_2_ receptor^11^, PTH receptor^12^, LH-RH receptor^13^, and oxytocin receptor^14^ were not significantly modified by the depletion of *N*-glycosylation, while those of rhodopsin^15^, β_2_-adrenergic receptor^16^, and angiotensin II type-1 receptor^17^ were all reduced, akin to Gpr176. Moreover, depending on the type of receptor examined, *N*-glycosylation can be either critical or non-critical for GPCR function. For example, *N*-glycosylation of the vasopressin V1a receptor is critical to maintain optimal ligand binding affinity^25^ and *N*-glycosylation of P2Y_12_ receptor is required to induce proper downstream G_i_-mediated signalling^26^. By contrast, *N*-glycosylation is not needed for the receptor functions of rhodopsin^15^, histamine H_2_ receptor^10^, and angiotensin II type-1 receptor^17^. Because of these versatile and non-uniform effects of *N*-glycosylation, it has been widely acknowledged that the role of the *N*-glycosylation of specific GPCRs must be determined empirically^23^. To the best of our knowledge, in this regard, Gpr176 is the first orphan GPCR whose *N*-glycosylation has been verified to be indispensable for proper protein expression. *N*-glycosylation is not essential for the molecular function of Gpr176. However, deficient *N*-glycosylation caused a drastic reduction in protein expression and thereby led to reduced total cAMP-repressive activity in the cells. Thus, *N*-glycosylation is likely a prerequisite for the proper protein expression of functional Gpr176.

Pharmacological treatment indicated that MG132, but not bafilomycin A1, partially rescued the expression of the non-glycosylated N-ter mut Gpr176, while expression of WT Gpr176 was not substantially increased by either MG132 or bafilomycin A1. We also observed accumulation of dysglycosylated Gpr176 in the ER, where GPCRs are normally synthesised and *N*-glycosylated^28–32^. These lines of circumstantial evidence suggest that the absence of *N*-glycan modification destabilizes this protein, possibly due to improper folding that renders the protein susceptible to ER-associated quality control leading to proteasomal degradation^28–32^. Further investigation will be necessary to understand the precise mechanism of reduced protein expression of Gpr176.

Finally, to begin to extend our findings to humans, we studied polymorphic variations of hGPR176. We found that two independent nonsynonymous SNPs located in the conserved N-terminal *N*-glycosylation sites of human GPR176, rs1473415441 and rs761894953, affect protein expression and cAMP-repressive activity in the cells. These two SNPs are both rare genetic variants (minor allele frequency < 0.01%) with no previously reported association with (patho)physiology. Thus, currently, physiological contribution of *N*-glycosylation of hGPR176 is completely unclear. Moreover, rare-variant effects cannot easily be identified via genome-wide association studies (GWAS) because of the problem of statistical power. In this sense, however, it is interesting to note that a previous GWAS of 89,283 individuals identified *ALG10B*, a gene encoding an enzyme catalysing the formation of *N*-glycan, as being associated with human circadian behaviour^37^. Although the mechanism by which ALG10B affects the circadian clock system is unknown, it is tempting to speculate that ALG10B might modify the extent of *N*-glycosylation of hGPR176. We found that Gpr176 is *N*-glycosylated in the mouse hypothalamic SCN, a structure known to function as the master clock of the body. In addition, based on amino acid sequence analysis, other circadian clock-related GPCRs and non-GPCR proteins that operate in the SCN (such as GPCR VPAC2 and the ion channel NMDA receptor) may be *N*-glycosylated *in vivo*. Therefore, examining whether ALG10B-mediated *N*-glycan modifications affect the *in vivo* physiological function and expression of Gpr176/GPR176 (and other GPCRs/non-GPCRs in the SCN) will be our next challenge.

## Methods

### Mouse hypothalamic membrane protein samples

All animal experiments were conducted in compliance with ethical regulations in Kyoto University and performed under protocols approved by the Animal Care and Experimentation Committee of Kyoto University. The brain hypothalamus was collected from C57BL/6 male WT mice or C57BL/6-backcrossed *Gpr176*^−/−^ mice^5^ and homogenized with a Dounce tissue grinder in a hypotonic buffer containing 20 mM HEPES (pH7.8), 2 mM EDTA, 1 mM DTT, and 1 × cOmplete Protease Inhibitor cocktail (Roche Diagnostics). After centrifugation at 20,400 × *g* for 30 min, the pellet was resuspended in a high-salt buffer containing 500 mM NaCl, 20 mM HEPES (pH7.8), 2 mM EDTA, 1 mM DTT, and 1 × cOmplete Protease Inhibitor cocktail. The mixture was then centrifuged, and the resultant pellet was solubilized with a detergent-containing buffer (20 mM HEPES [pH7.8], 150 mM NaCl, 2 mM EDTA, 1 mM DTT, 1% cholesteryl hemisuccinate, 0.2% dodecyl-β-d-maltoside, and 1 × cOmplete Protease Inhibitor). The soluble fractions were either subjected to glycosidase treatment (see below for details) or denatured in Laemmli buffer for immunoblot analysis. All procedures were carried out at 4 °C.

### Flp-In TREx 293 cell cultures and treatments

Flp-In TREx293-Gpr176 (tet-on) cells were generated by stable transfection of Flp-In T-REx 293 cells (Thermo Fisher Scientific) with a pcDNA5/FRT vector containing the untagged full-length coding sequence of the mouse *Gpr176* (NM_201367). Point mutations at the potential *N*-glycosylation sites (N4, N11, N17, N26, N295) were generated with a standard sequential PCR method^38^. To generate Gpr176-GFP, the entire coding sequence of Gpr176 without stop codon was fused in frame to the N-terminus of GFP. To create Flp-In TREx293-Gpr176 (tet-on)/GloSensor (constitutive) cells, we constructed a modified pcDNA5/FRT vector carrying Gpr176 and GloSensor-22F (Promega) under different promoters: while *Gpr176* was cloned into a proprietary pcDNA5/FRT cloning site for tet-on induction, *GloSensor* was cloned separately into a different position of the vector (at a unique *Pci*I site) in conjunction with a tet-insensitive CMV promoter. Cells were cultured, unless otherwise specified, at 37 °C under 5% CO_2_ in DMEM medium (Nacalai, #08458-16) containing 10% fetal bovine serum, 100 µg/ml hygromycin, 10 µg/ml blasticidin, and 1% Antibiotic-Antimycotic Mixed solution (Nacalai). For Dox treatment, doxycycline (Clontech Laboratories) was added to the medium to a final concentration of 1 µg/ml. Where indicated, MG132 (Calbiochem) or bafilomycin A1 (Sigma) was added to the culture medium after 15 h of Dox treatment. For human SNP analysis, Flp-In TREx293 clonal cell lines were generated with a full-length coding sequence of the human *GPR176* (NM_007223), which was obtained from Genscript (OHu08024D).

### Glycosidase treatment

PNGase F and *O*-glycosidase were purchased from New England BioLabs and used according to the manufacturer’s instructions. Flp-In TREx293 (tet-on) cells or mouse hypothalamus cell membranes were incubated with a mixture of cholesteryl hemisuccinate (1%) and dodecyl-β-d-maltoside (0.2%), and their soluble fractions were denatured using 1 × Glycoprotein Denaturing Buffer (New England BioLabs) for 5 min at 70 °C. After chilled on ice, the denatured proteins were incubated with PNGase F (50 U/µl) or *O*-glycosidase (400 U/µl) in 1 × G7 Reaction Buffer (New England BioLabs) supplemented with 1% NP-40 for 2 h at 37 °C. The reaction was stopped by Laemmli buffer for the subsequent analysis.

### Immunoblot

Immunoblotting was performed using our standard method^39^ with affinity-purified antibody against Gpr176. Gpr176 antiserum was acquired in rabbit using a glutathione-*S*-transferase (GST)-fused Gpr176 mouse protein fragment (amino acids (a.a.) 311–515)^5^. The affinity-purified Gpr176 antibody used in this study was re-prepared from the same batch of the antiserum using a maltose-binding protein (MBP)-fused Gpr176 fragment (a.a. 311–515). Antibodies for β-Actin (Sigma, A5441) and Hsp70 (Santa Cruz, sc-66048) were also used for internal control. Uncropped blots are available in Supplementary Fig. 8.

### RNA extraction and qRT-PCR

Total RNA was extracted with RNeasy kit (Qiagen) and converted to cDNA with SuperScript VILO cDNA Synthesis kit (Thermo Fisher Scientific). Real-time PCR was performed using THUNDERBIRD SYBR qPCR Mix (TOYOBO) on a StepOnePlus Real-Time PCR System (Thermo Fisher Scientific). Control primers for *RPLP0* (NM_053275) were F (5′-ATG CAG CAG ATC CGC ATG T-3′) and R (5′-TTG CGC ATC ATG GTG TTC TT-3′), and primers for *Gpr176* (NM_201367) were F (5′-CAT CTT CAT TGG CTC GCT AC-3′) and R (5′- CGT ATA GAT CCA CCA GCA AC-3′).

### Fluorescence microscopy

Flp-In TREx293 Gpr176-GFP (tet-on) cells and the related cells that express an N-terminal *N*-glycosylation mutant Gpr176-GFP were plated on polylysine-coated coverslips in culture medium containing 1 µg/ml Dox for 15 h. The cells were fixed with 4% paraformaldehyde and mounted in ProLong Gold antifade reagent with DAPI (Thermo Fisher Scientific). The Gpr176 GFP fusion protein subcellular localization was monitored using direct fluorescence from the GFP moiety. For ER staining, cells were treated with 1 μM ER-Tracker Red (Thermo Fisher Scientific). ER-Tracker is a fluorescence-labeled glibenclamide that is capable of visualizing ER via specific binding to the sulphonylurea receptors of ATP-sensitive K^+^ channels, which are prominent on ER^40^. For calnexin immunocytochemistry, cells were fixed, permeabilized, and blocked with 5% bovine serum albumin in PBS containing 0.1% Triton X-100, as described^41^. The cells were immunolabeled with anti-calnexin (BD Transduction Laboratories, #610523) and visualized with Alexa594-conjugated anti-mouse IgG (Thermo Fisher Scientific). Images were captured using an Olympus FV10I-DOC confocal microscope.

### GloSensor-cAMP assay

Flp-In TREx293-Gpr176 (tet-on)/GloSensor (constitutive) cells and the related cells that express an *N*-glycosylation-deficient Gpr176 were seeded in a collagen I-coated 96-well plate (Corning) at a density of 5.4×10^4^ cells per well with a carbon dioxide-independent DMEM medium (Sigma, D2902) containing 10% bovine serum, 0.035% sodium bicarbonate, 10 mM HEPES (pH 7.2), 3.5 g/l D-glucose, and 1% Antibiotic-Antimycotic Mixed solution (Nacalai). D-luciferin (Promega) was also included in the medium at a concentration of 1 mM. After 6 h at 37 °C, the cells received Dox (final, 1 µg/ml) or vehicle and underwent additional incubation at 37 °C for 15 h. Prior to luminescence detection, the cell culture plate was acclimatized to 27 °C for 1 h. Luminescence was then recorded on a FDSS/μCELL plate reader (Hamamatsu Photonics) at 27 °C every 5 sec. Forskolin (final, 10 μM) was added to the culture medium 2 minutes after the start of measurement. Data were integrated over 1-min or 30-sec intervals, and the values were normalized to the average peak luminescence of Dox (−) cells.

For the verification of intracellular total GloSensor levels in Dox-treated and non-treated cells, cells were lysed into a passive lysis buffer (Promega) with a volume of 100 μl per well, and aliquots of the lysates were mixed with or without cAMP (final, 1 or 4 µM) and assayed for GloSensor activity using a Luciferase Assay Reagent II (Promega).

### cAMP enzyme immunoassay

Intracellular cAMP levels were determined by EIA. Cells were lysed into 6% perchloric acid solution containing 4 mM theophylline (Sigma) at 4 °C and neutralized with 0.72 M KOH/0.6 M KHCO_3_. After removal of salt precipitants, the extracts were subjected to an enzyme immunoassay for cAMP using a kit purchased from Cayman Chemical (Cat. No 581001)^5,42^.

### Glycosidase-treated cell suspension GloSensor assay

Cells of the same batch were cultured in parallel with or without Dox (1 µg/ml) for 15 h and then removed from dish with Versene solution (Life Technologies). Cells were dissociated into single cells through gentle trituration and filtered via a 70-μm cell strainer (BD Falcon). Cells were resuspended in HBSS containing 5 mM HEPES (pH7.5) and 0.1% bovine serum albumin at a cell density of 8 × 10^6^ cells/ml. The suspensions were then immediately treated with PNGase F (5 U/μl) or vehicle for 1 h at 37 °C with gentle agitation. Following enzymatic depletion of *N*-linked glycosylation, cells were transferred into fresh HBSS containing 5 mM HEPES (pH7.5), 0.1% bovine serum albumin, and 1 mM luciferin, and cells in suspension (~1×10^5^ cells per well) were assayed for GloSensor activity. Data were normalized with total GloSensor levels, for which cell lysates were incubated with excess cAMP.

## Supplementary information

Supplementary information.

## Data Availability

The datasets generated and analyzed during the current study are available from the corresponding author on reasonable request.
