# Supplementary material for: Identification and functional characterisation of N-linked glycosylation of the orphan G protein-coupled receptor Gpr176
Source: Sci Rep. 2020 Mar 10;10:4429. doi: 10.1038/s41598-020-61370-y (PMC7064540; doi:10.1038/s41598-020-61370-y)
Supplement: Supplementary file 1 — Supplementary information. [file 41598_2020_61370_MOESM1_ESM.pdf]

Supplementary Information

Identification and functional characterisation of *N*-linked glycosylation of the orphan G protein-coupled receptor Gpr176

Tianyu Wang[1†], Shumpei Nakagawa[1†], Takahito Miyake[1], Genzui Setsu[1], Sumihiro Kunisue[1], Kaoru Goto[1], Akira Hirasawa[2], Hitoshi Okamura[1,3], Yoshiaki Yamaguchi[1], Masao Doi [1,*]

[1]Department of Systems Biology, Graduate School of Pharmaceutical Sciences, Kyoto University, Sakyō-ku, Kyoto 606-8501, Japan.
[2]Department of Genomic Drug Discovery Science, Graduate School of Pharmaceutical Sciences, Kyoto University, Sakyō-ku, Kyoto 606-8501, Japan.
[3]Present address: Laboratory of Molecular Brain Science, Graduate School of Pharmaceutical Sciences, Kyoto University, Sakyō-ku, Kyoto 606-8501, Japan.

[†]T.W. and S.N. contributed equally to this work.

[*]Address correspondence to:
Masao Doi, doimasao@pharm.kyoto-u.ac.jp

Supplementary Information: Supplementary Figures 1, 2, 3, 4, 5, 6, 7, and 8.

Supplementary Figure 1

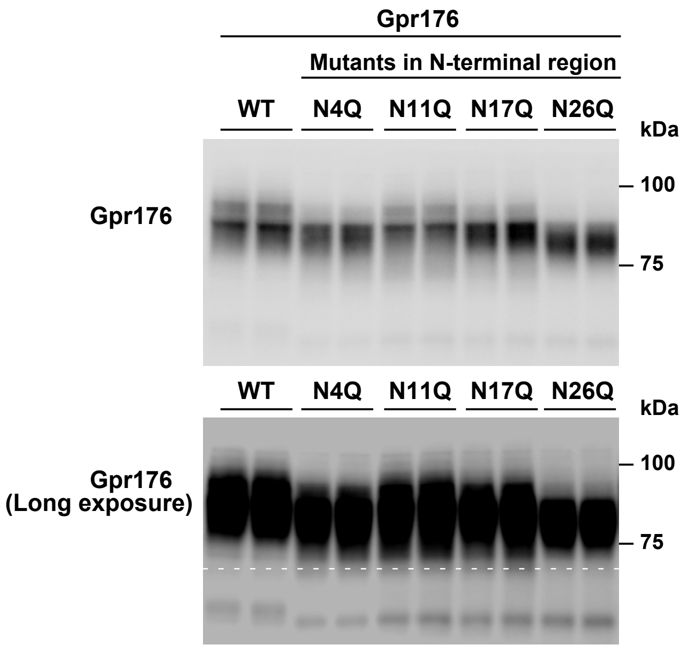

Supplementary Figure 1 | Comparison of SDS–PAGE migration profiles of wild-type (WT) Gpr176 and mutants containing a single Asn (N) to Gln (Q) substitution of the potential N-terminal glycosylation sites. The WT Gpr176 and respective N4Q, N11Q, N17Q, and N26Q mutants were separately expressed in Flp-In TREx293 cells and subjected to SDS–PAGE/immunoblot analysis with anti-Gpr176 antibody. A longer exposure of the same immunoblot is shown on the bottom. A dashed white horizontal line is overlaid on the high exposure images to emphasize the differences in size between WT and mutants. The WT Gpr176 was recognized as a broad band with a molecular weight slightly higher than 75 kDa. The broadness of the band implies *N*-linked glycosylation heterogeneity. All tested mutants exhibited a small but noticeable change in mobility, with N4Q and N26Q evoking larger mobility changes than N11Q and N17Q. These results suggest that all four N-terminal consensus sites are glycosylated.

Supplementary Figure 2

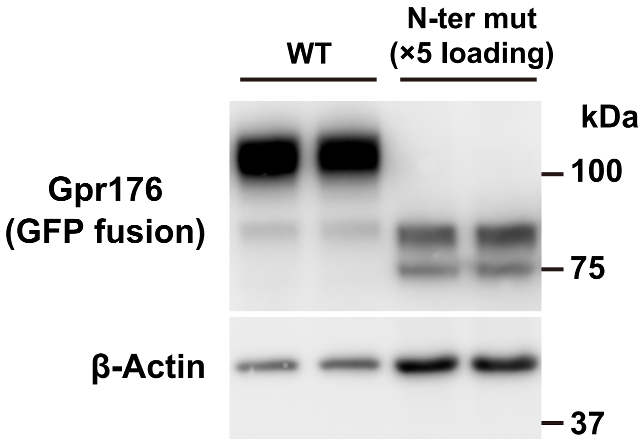

Supplementary Figure 2 (related to Fig. 3) | *N*-glycosylation deficiency leads to decreased expression of GFP-fused Gpr176. Flp-In TREx293 cells expressing either WT or N-ter mut Gpr176-GFP fusion protein were immunoblotted for Gpr176 (upper) and β-Actin (lower). The protein extracts from N-ter mut cells were loaded five-fold over WT cells to increase the sensitivity of protein detection.

Supplementary Figure 3

N-ter mut
Gpr176-GFP

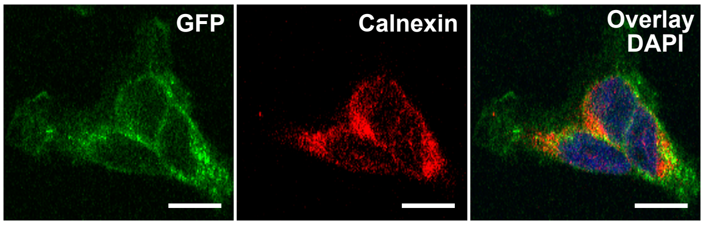

Supplementary Figure 3 (related to Fig. 3) | Partially overlapped localization of N-ter mut Gpr176 and calnexin in Flp-In TREx293 cells. Cells expressing N-ter mut Gpr176-GFP (green) were immunolabeled for calnexin (red). Calnexin is an ER-resident lectin chaperone that binds with *N*-linked oligosaccharides containing terminal glucose residues. The merged image is a combined image with DAPI (blue). Cells are representative of a population with independent experiments. Scale bars, 10 µm. Note that in the ER, calnexin (red) and N-ter mut Gpr176 (green) were not completely overlapped, likely consistent with the idea that N-ter mutation of Gpr176 results in the loss of *N*-glycans to which calnexin binds.

Supplementary Figure 4

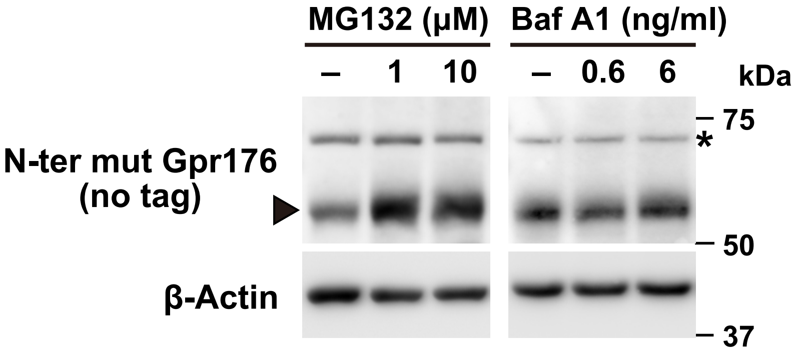

Supplementary Figure 4 (related to Fig. 3) | Increased expression of glycosylation-deficient Gpr176 by treatment with MG132 but not with bafilomycin A1. In this assay, we analyzed non-tagged Gpr176 (i.e., no C-terminal GFP). Flp-In TREx293 cells that express *N*-glycosylation deficient Gpr176 (i.e., N-ter mut) were treated with either MG132 or bafilomycin A1 at the indicated concentrations for 6 hours and immunoblotted for Gpr176 (upper) and β-Actin (lower). Arrowhead indicates the position of aglycosylated Gpr176. Asterisk, a non-specific band (see **Fig. 2B**).

Supplementary Figure 5

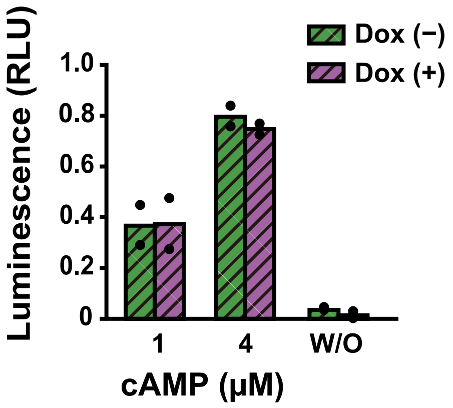

Supplementary Figure 5 (related to Fig. 4) | Comparable basal GloSensor levels between Dox-treated (+) and non-treated (−) cells. The GloSensor system relies on the biosensor expression. To be sure that the intracellular functional GloSensor level does not differ between Dox-treated and non-treated cells, we verified whether their cell lysates are able to show similar GloSensor activities when incubated in vitro with fixed amounts of cAMP. The cells were lysed with or without (w/o) standard cAMP (final conc.: 1 or 4 μM) in buffer containing D-luciferin, Mg^{2+}, and ATP. Luminometry showed that both cell lysates exhibit equivalent dose-dependent GloSensor activities, providing evidence that Dox treatment does not affect GloSensor expression in the cell. Data shown are biological replicates ($n = 2$, for each condition).

Supplementary Figure 6

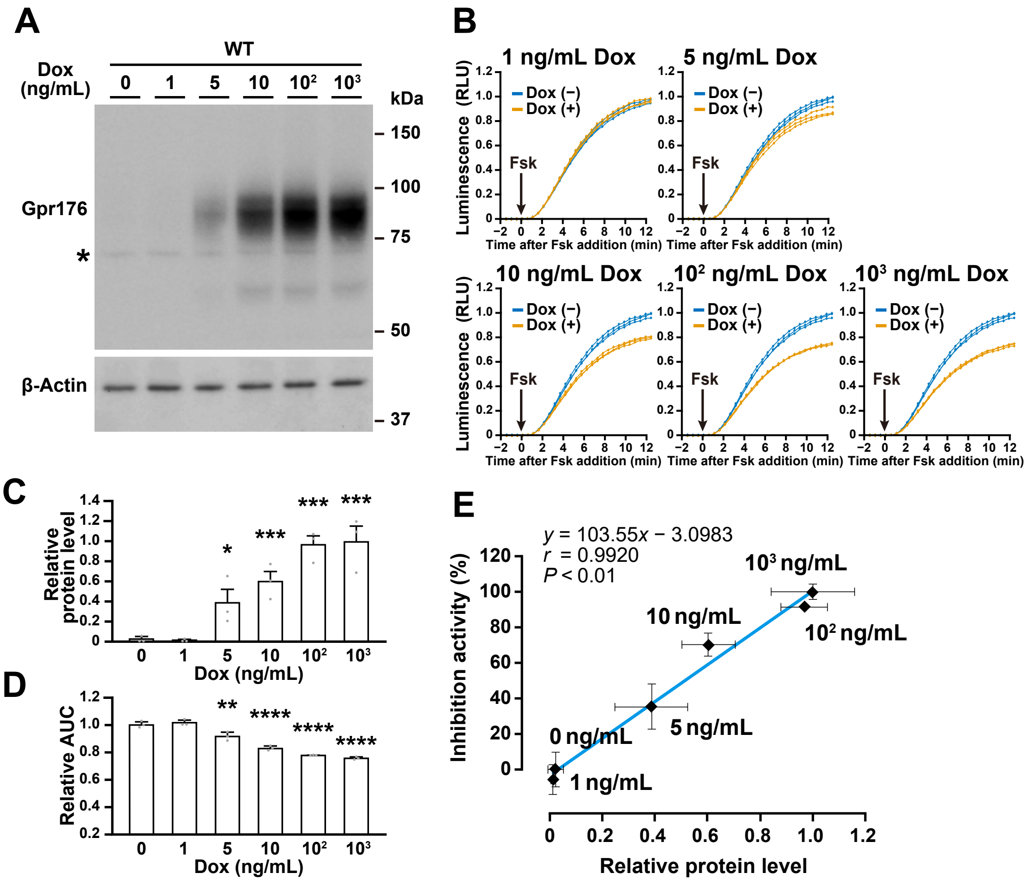

Supplementary Figure 6 (related to Fig. 4) | Correlation between Gpr176 protein expression level and its cellular cAMP-repressing activity. (**A**) Immunoblot of Flp-In TREx293-Gpr176 (tet-on) cells. Cells were treated with increasing doses of Dox (0. 1, 5, 10, 10^2, and 10^3 ng/mL) and immunoblotted for Gpr176 (upper) and β-Actin (lower). Asterisk, nonspecific bands. (**B**) GloSensor activity traces of Flp-In TREx293-Gpr176 (tet-on) cells. Cells were treated with increasing doses of Dox (0. 1, 5, 10, 10^2, and 10^3 ng/mL). For comparison, GloSensor traces of Dox untreated cells (i.e., 0 ng/mL) are displayed in parallel. Data represent three independent biological replicates per condition. RLU, relative light units. (**C**) Relative protein expression levels of Gpr176. Values are the means ± s.d. (n = 3 for each) of the relative band intensities in (**A**). *P<0.05, ***P<0.005, versus Dox-untreated cells, one-way ANOVA with Bonferroni *post hoc* test. (**D**) Relative area under the curve (AUC) of luminescence values in (**B**). Light emissions were integrated and normalized with those of the untreated control. **P<0.001, ****P<0.0001, versus Dox-untreated cells, one-way ANOVA with Bonferroni *post hoc* test (n = 3 for each condition). Error bars indicate s.d. (**E**) Relationship between protein expression level (x axis) and inhibition activity (y axis) of Gpr176 in cells treated with different doses of Dox. Values are the means ± s.d. (n = 3 for each). Correlation coefficient (r) and P value were calculated by Pearson product moment correlation coefficient analysis (r = 0.9920, P<0.01). The linear regression equation is shown at the top of figure.

Supplementary Figure 7

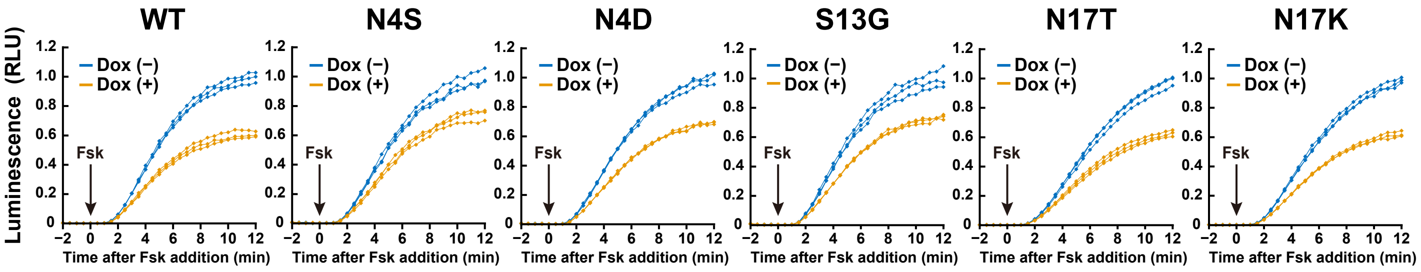

Supplementary Figure 7 (related to Fig. 6) | GloSensor activity traces in Dox-treated (+) and untreated (−) Flp-In TREx293 cells expressing WT hGPR176 and its variants N4S, N4D, S13G, N17T, and N17K. Data represent three independent biological replicates per condition. RLU, relative light units. Values are plotted relative to the average peak value obtained in untreated cells. Relative area under the curve (AUC) values were used to assess the extent of cAMP-inhibitory activity of each hGPR176 variant (see **Fig. 6I**).

Supplementary Figure 8

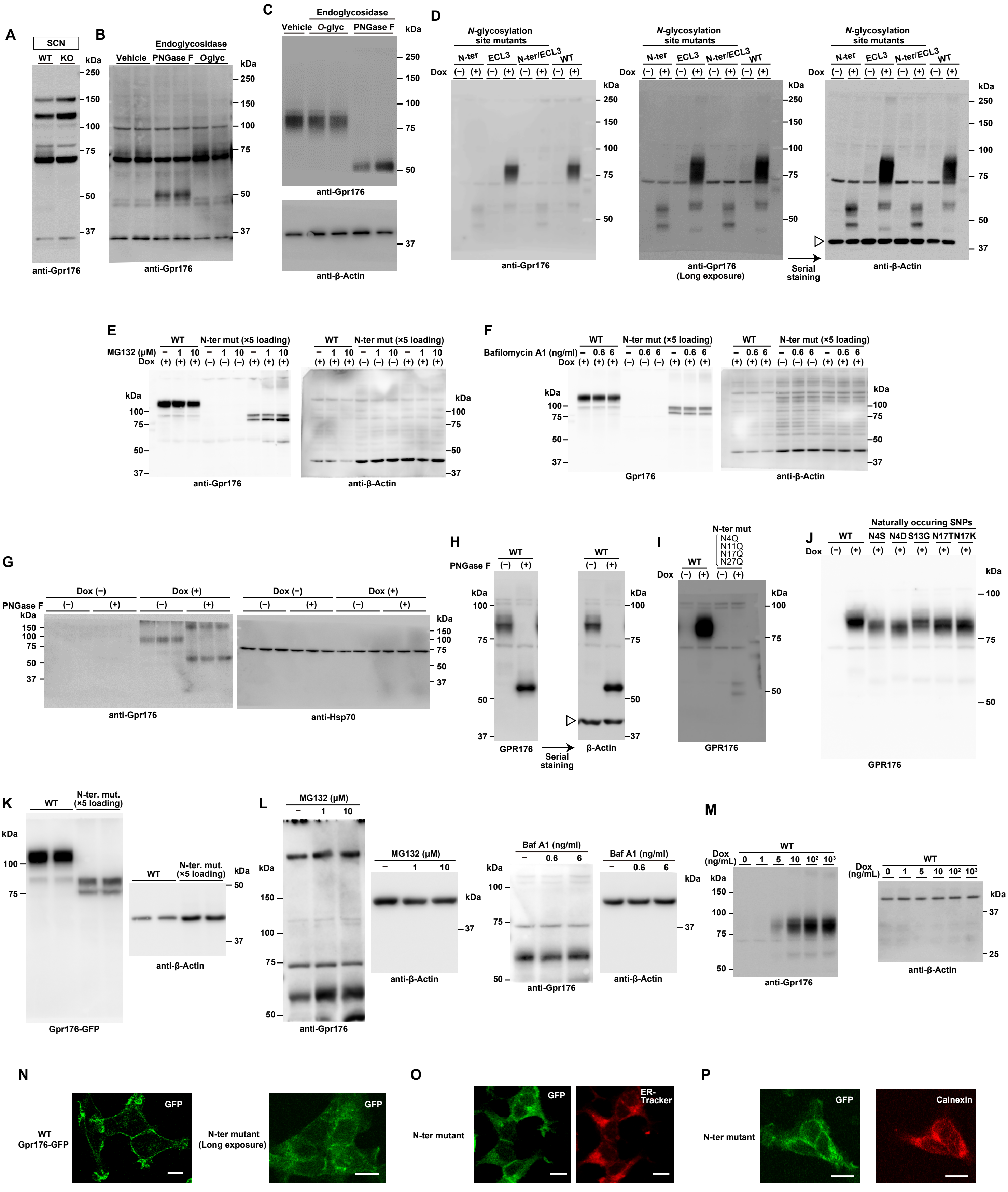

Supplementary Figure 8 | Full version of Western blots shown in Fig. 1B (**A**), Fig. 1C (**B**), Fig. 2A (**C**), Fig. 2B (**D**), Fig. 3C (**E**), Fig. 3D (**F**), Fig. 5C (**G**), Fig. 6B (**H**), Fig. 6C (**I**), Fig. 6G (**J**), Supplementary Fig. 2 (**K**), Supplementary Fig. 4 (**L**), Supplementary Fig. 6A (**M**), and unaltered confocal images shown in Fig. 3A (**N**), Fig. 3B (**O**), and Supplementary Fig. 3 (**P**).
